# Supplementary material for: The interactome of intact mitochondria by cross-linking mass spectrometry provides evidence for coexisting respiratory supercomplexes
Source: Mol Cell Proteomics. 2017 Dec 8;17(2):216–32. doi: 10.1074/mcp.RA117.000470 (PMC5795388; doi:10.1074/mcp.RA117.000470)
Supplement: Supplemental Data [file supp_RA117.000470_133922_0_supp_23978_fzffwf.zip › spectra_annotation/mito_spectra_annotation/122-1-3-1-4-1.pdf]

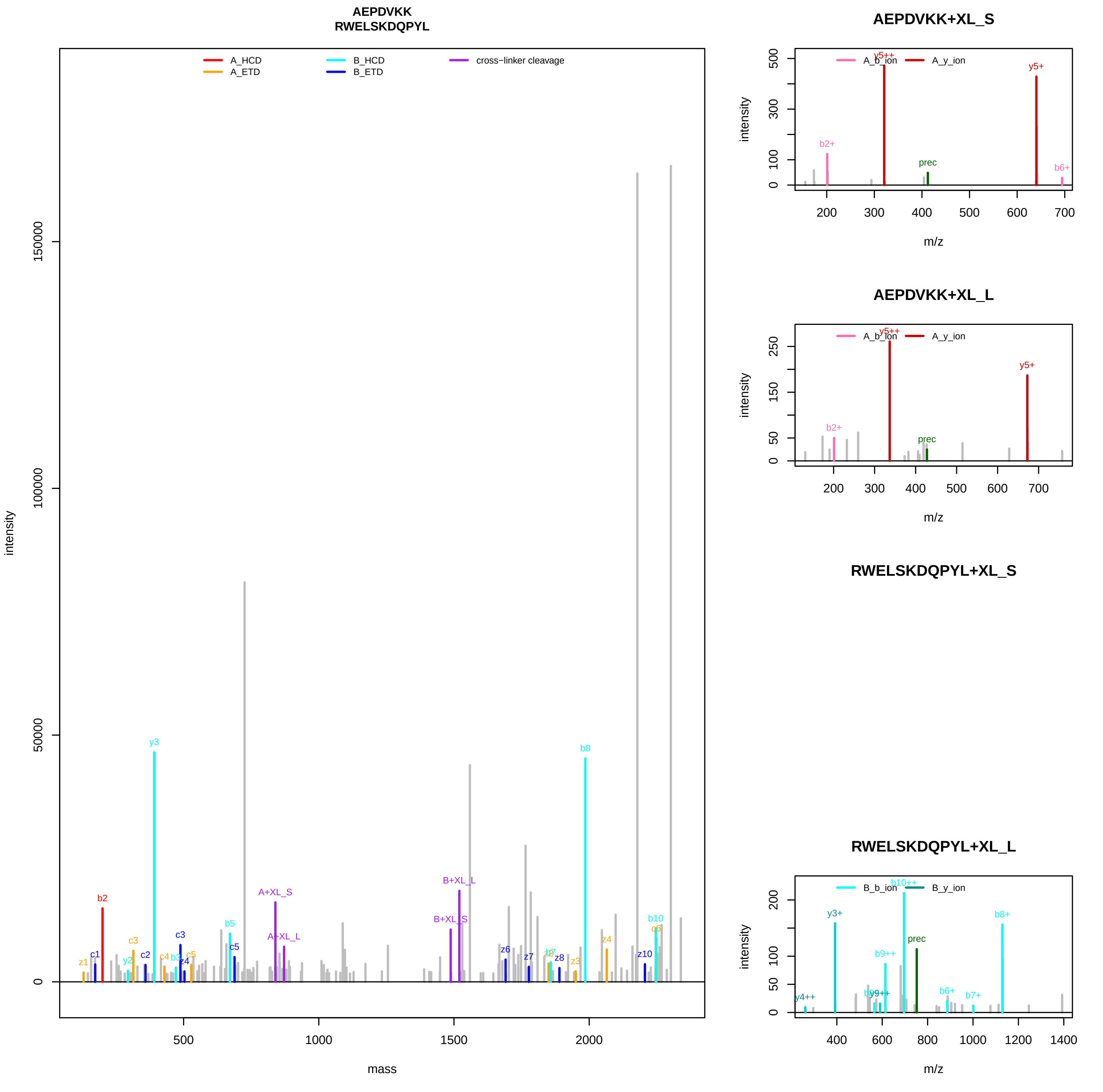

AEPDVKK
RWELSKDQPYL
A_HCD
A_ETD
B_HCD
B_ETD
cross−linker cleavage
intensity
mass
0
50000
100000
150000
500
1000
1500
2000
z1
c1
b2
y2
c3
c2
c4
b3
z4
c3
c5
b5
c5
A+XL_S
A+XL_L
B+XL_S
B+XL_L
z6
z7
z8
z3
b8
z4
z10
b10
c6
y3
AEPDVKK+XL_S
A_b_ion
A_y_ion
y5++
y5+
b2+
prec
b6+
intensity
m/z
0
100
300
500
200
300
400
500
600
700
AEPDVKK+XL_L
A_b_ion
A_y_ion
y5++
y5+
b2+
prec
intensity
m/z
0
50
150
250
200
300
400
500
600
700
RWELSKDQPYL+XL_S
RWELSKDQPYL+XL_L
B_b_ion
B_y_ion
b10++
y3+
b8+
prec
b9++
y4++
y9++
b6+
b7+
intensity
m/z
0
50
100
200
400
600
800
1000
1200
1400
